# Molecular profiling of clinical remission in psoriatic arthritis reveals dysregulation of *FOS* and *CCDC50* genes: a gene expression study

**DOI:** 10.3389/fimmu.2023.1274539

**Published:** 2023-10-27

**Authors:** Maria Maddalena Angioni, Alberto Floris, Ignazio Cangemi, Mattia Congia, Elisabetta Chessa, Micaela Rita Naitza, Matteo Piga, Alberto Cauli

**Affiliations:** ^1^ Department of Medical Science and Public Health, University of Cagliari, Cagliari, Italy; ^2^ Rheumatology Unit, Azienda Ospedaliero - Universitaria di Cagliari, Cagliari, Italy

**Keywords:** psoriatic arthritis, clinical remission, transcriptomic, *FOS*, *CCDC50*

## Abstract

**Background:**

In psoriatic arthritis (PsA), the primary goal of treatment is clinical remission. This study aimed to characterize the molecular profile underlying the induced clinical remission in patients with PsA, comparing the remission state and the healthy condition.

**Methods:**

Whole blood transcriptomic analysis was performed on groups of 14 PsA patients in TNFi-induced clinical remission (DAPSA ≤ 4), 14 PsA patients with active disease (DAPSA > 14), and 14 healthy controls (HCs). Then, all differentially expressed genes (DEGs) derived from remission vs. HC comparison were analyzed for functional and biological characteristics by bioinformatics software. The gene expression of 12 genes was then validated by RT-qPCR in an extended cohort of 39 patients in clinical remission, 40 with active disease, and 40 HCs.

**Results:**

The transcriptomic analysis of PsA remission vs. HCs highlighted the presence of 125 DEGs, and out of these genes, 24 were coding genes and showed a great involvement in immune system processes and a functional network with significant interactions. The RT-qPCR validation confirming the down- and upregulation of *FOS* (FC −2.0; *p* 0.005) and *CCDC50* (FC +1.5; *p* 0.005) genes, respectively, in line with their role in orchestrating inflammation and bone metabolism processes, may be related to PsA pathophysiology.

**Conclusion:**

The transcriptomic profile of clinical remission in PsA is similar to a healthy condition, but not identical, differing for the expression of *FOS* and *CCDC50* genes, which appears to play a key role in its achievement.

## 1 Introduction

Psoriatic arthritis (PsA) is a chronic inflammatory disease characterized by wide clinical heterogeneity due to the variable combination of six major domains, namely, skin and nail psoriatic lesions, peripheral arthritis, axial disease, dactylitis, and enthesitis (1). It is recognized as a potentially disabling disease, as late and inadequate control of disease activity may result in structural damage and disability (2).

According to the European Alliance of Associations for Rheumatology (EULAR) and the Group for Research in Psoriasis and PsA (GRAPPA) recommendations, treatment of PsA should aim primarily at reaching the target of remission by regular disease activity assessment and appropriate adjustment of therapy (3, 4). Although this approach represents one of the strongest and most widely shared recommendations, there are still relevant issues regarding its application in clinical practice. In particular, the definition of remission is still open to discussion among experts and represents a significant challenge in the management of PsA (5). Several definitions of clinical remission, based on composite indices combining objective (e.g., tender and swollen joint count or enthesitis and dactylitis count (6)) and subjective (e.g., scales for pain or general health) measurements of disease activity are currently used in clinical practice and trials (7). However, the clinical heterogeneity of PsA, the potential persistence of subclinical disease activity demonstrated in ultrasonography studies, and the possible progression of structural damage in patients classified as in clinical remission (8), highlight the urgent need for a sensitive and specific biomarker supporting the accurate identification of remission.

Several genetic, circulating, and tissue factors have been studied as biomarkers in the management of different aspects of PsA, including diagnosis and assessment or prediction of disease activity, severity, and response to treatment (9–13). However, none of these has been extensively validated and then translated into routine clinical practice (10, 14). In particular, despite remission being recommended as the primary goal in PsA treatment, to our knowledge, no studies have been specifically designed to identify the underlying molecular mechanisms and potential biomarkers.

Transcriptomic profiling has become a standard technology in searching for biomarkers of susceptibility, disease activity, progression, and response to treatment in several diseases, including PsA (15, 16). However, the transcriptomic approach has yet to be applied so far in the assessment of clinical remission. Since sustained clinical remission without drug treatment is extremely rare in patients with PsA, a substantial molecular difference between clinical remission and the healthy state may be assumed, but it needs to be demonstrated and characterized. In this regard, an intriguing question is whether the achievement of clinical remission reflects a molecular profile closer to healthy individuals rather than PsA active patients, which we refer to as “molecular remission”.

This study aimed to identify molecular remission biomarkers by comparing the gene expression profile of PsA patients in clinical remission vs. healthy controls and PsA patients with active disease.

## 2 Methods

### 2.1 Patients and controls

The present study was based on the comparative transcriptomic profiling of three groups of subjects: PsA patients in clinical remission for at least 1 year (PsA-R), PsA patients with active disease (PsA-A), and healthy controls (HCs). The PsA patients, recruited from a monocentric cohort, were diagnosed according to the classification criteria for psoriatic arthritis (CASPAR) (17) and classified as in clinical remission or active if they had a Disease Activity PsA (DAPSA) score of ≤4 or >14, respectively (18). To ensure a higher level of homogeneity of the PsA group in clinical remission, all the recruited patients were on treatment with TNF inhibitors (TNFi) after the failure of methotrexate. The treatment regimen of the PsA group with active disease is reported in **Table 1**. Patients undergoing concomitant treatment with glucocorticoids were excluded from both groups. The healthy control group was matched for mean age and gender ratio with the remission group, as this study was primarily focused on comparing these two conditions.

**Table 1:** Demographic, clinical, and therapeutic data of the recruited PsA patients in clinical remission (PsA-R), PsA patients with active disease (PsA-A), and healthy controls (HCs).

	PsA-R (*n* = 39)	PsA-A (*n* = 40)	HC (*n* = 40)
Demographics			
Male, *n* (%)	30 (76.9)	20 (50.0)	19 (47.5)
Age at enrolment, mean (SD), years	52.0 (12.3)	55.5 (14.9)	52.0 (6.3)
Disease duration, mean (SD), years	10.1 (6.3)	5.6 (5.6)	–
BMI, mean (SD) score	25.5 (3.7)	27.7 (4.9)	
Clinical pattern			
Axial, *n* (%)	11/39 (28.2)	1/40 (2.5)	–
Peripheral, *n* (%)	39/39 (100)	40 (100)	–
Personal history of PsA (%)	37/39 (94.9)	39/40 (97.5)	
Familiar history of PsA (%)	6/38 (15.6)	5/40 (12.5)	
Onychopathy (%)	22/37 (59.7)	22/38 (57.9)	
Dactylitis (%)	26/39 (66.7)	21/40 (52.5)	
Enthesitis (%)	23/38 (60.5)	17/40 (42.5)	
Rheumatoid factor, *n* (%)	8/39 (20.5)	1/34 (2.9)	–
Clinimetrics			
PGA, mean (SD), years	2.9 (7.6)	45.8 (28.0)	–
PtGA, mean (SD), years	15.3 (19.9)	69.0 (20.9)	–
VAS—pain, mean (SD), years	15.1 (21.3)	70.4 (18.2)	–
GH, mean (SD), years	75.2 (21.6)	51.8 (25.4)	–
ESR, mean (SD), years	9.6 (6.4)	29.1 (19.9)	–
CRP, mean (SD), years	1.1 (1.2)	12.5 (22.1)	–
DAS-28, mean (SD), years	1.9 (0.7)	4.6 (1.3)	–
DAPSA, mean (SD), years	3.6 (4.9)	25.8 (10.4)	–
HAQ, mean (SD), years	0.4 (0.4)	1.4 (0.6)	–
Treatment			
NSAID, *n* (%)	11 (28.2)	13 (32.5)	–
cs-DMARDs, *n* (%)	10 (25.6)	23 (57.5)	–
TNF inhibitors, *n* (%)	39 (100)	7 (17.5)	–

BMI, body mass index; PGA, Physician Global Assessment; PtGA, Patient Global Assessment, VAS, visual analog scale; GH: Global Health Assessment; ESR: erythrocyte sedimentation rate; CRP, C-reactive protein; DAS-28, Disease Activity Score-28; DAPSA, Disease Activity Index for Psoriatic Arthritis; HAQ, Health Assessment Questionnaire; NSAIDs, non-steroidal anti-inflammatory drugs; csDMARDs, conventional synthetic disease-modifying antirheumatic drugs. - means NONE.

The demographic and clinical features of the three study groups are reported in **Table 1**.

The study was approved by the local ethical committee (PG/2018/16313; 12th November 2018), and written informed consent was obtained from all subjects. All procedures were in accordance with the Good Clinical Practice standards and Helsinki Declaration.

### 2.2 Study design

The study consisted of three consecutive phases:

I. *Explorative transcriptomic profiling*: To identify a preliminary list of differentially expressed genes (DEGs), transcriptomic analysis was performed on pooled RNAs from peripheral blood in biological duplicates of a group of 14 PsA patients in clinical remission, 14 PsA patients with active disease, and 14 HCs (groups of 7 patients in biological duplicates for each condition, for a total of 6 microarrays).

II. *Functional and biological analysis of dysregulated transcripts*: First, the complete list of DEGs identified by comparing the PsA-R vs. HC groups were analyzed *in silico* for functional and biological characteristics. Then, only mRNAs related to coding genes were selected and re-analyzed *in silico* to select those of greater interest to be assessed in the validation phase.

III. *RT-qPCR validation analysis*: A quantitative reverse transcription PCR (RT-qPCR) for single gene expression analysis of DEGs selected from the previous phases was extended in the whole cohort of 39 PsA patients in clinical remission, 40 PsA with active disease, and 40 HCs.

### 2.3 Transcriptomic analysis

#### 2.3.1 Target preparation

RNAs were extracted from peripheral blood in RNAlater preservative (Invitrogen) by Ambion RiboPure Kit followed by DNAse treatment. The quality of total RNA was assessed using an Agilent Bioanalyzer 2100 (Agilent Technologies, Palo Alto, CA, USA). Extracted RNAs were pooled in groups of seven patients in biological duplicates for each condition (remission, active, healthy controls) for a total of six microarrays.

Biotin-labeled cDNA targets were synthesized starting from 150 ng of total RNA. Double-stranded cDNA synthesis and related cRNA were performed with GeneChip^®^ WT Plus Kit (Affymetrix, Santa Clara, CA, USA). With the same kit, the sense strand cDNA was synthesized before being fragmented and labeled. All steps of the labeling protocol were performed as suggested by Affymetrix. Each eukaryotic GeneChip^®^ probe array contains probe sets for several *Bacillus subtilis* genes that are absent in the samples analyzed (lys, phe, thr, and dap). This Poly-A RNA Control Kit contains *in-vitro* synthesized, polyadenylated transcripts for the *B. subtilis* genes that are premixed at staggered concentrations to allow GeneChip^®^ probe array users to assess the overall success of the assay. The Poly-A RNA Control final concentrations in each target are as follows: lys, 1:100,000; phe, 1:50,000; thr, 1:25,000; and dap, 1:6,667.

#### 2.3.2 DNA microarray hybridization

This was performed using the GeneChip^®^ Hybridization, Wash and Stain Kit. It contains a mix for target dilution, DMSO at a final concentration of 7%, and premixed biotin-labeled control oligo B2 and bioB, bioC, bioD, and cre controls (Affymetrix cat. #900299, Santa Clara, CA, USA) at a final concentration of 50 pM, 1.5 pM, 5 pM, 25 pM, and 100 pM, respectively. Targets were diluted in a hybridization buffer at a 25-ng/μL concentration and denatured at 99°C for 5 min, incubated at 45°C for 5 min, and centrifuged at maximum speed for 1 min before introduction into the GeneChip^®^ cartridge. A single GeneChip^®^ Human Transcriptome Array 2.0 was then hybridized with each biotin-labeled sense target. Hybridizations were performed for 16 h at 45°C in a rotisserie oven. GeneChip^®^ cartridges were washed and stained with the GeneChip^®^ Hybridization, Wash and Stain Kit in the Affymetrix Fluidics Station 450 following the FS450_0002 standard protocol, including the following steps: 1) (wash) 10 cycles of 2 mixes/cycle with Wash Buffer A at 30°C; 2) (wash) 6 cycles of 15 mixes/cycle with Wash Buffer B at 50°C; 3) stain of the probe array for 5 min in SAPE solution at 35°C; 4) (wash) 10 cycles of 4 mixes/cycle with Wash Buffer A at 30°C; 5) stain of the probe array for 5 min in antibody solution at 35°C; 6) stain of the probe array for 5 min in SAPE solution at 35°C; 7) (final wash) 15 cycles of 4 mixes/cycle with Wash Buffer A at 35°C; and 8) fill the probe array with Array Holding buffer.

#### 2.3.3 Image acquisition, data processing, and bioinformatics analysis

GeneChip arrays were scanned using an Affymetrix GeneChip^®^ Scanner 3000 7G (Affymetrix, Santa Clara, CA, USA) using default parameters. Affymetrix GeneChip^®^ Command Console Software (AGCC) was used to acquire GeneChip^®^ images and generate.DAT and.CEL files, which were used for subsequent analysis with proprietary software (Partek Genomics suite V6.6).

To identify differentially expressed transcripts (concordantly on both biological duplicates of each profiled condition), a fold change (FC) ± 1.5 cutoff and a *p*-value of 0.05 were set.

### 2.4 Functional and biological analysis of dysregulated transcripts

For the bioinformatics Gene Ontology (GO) analysis, only differentially expressed transcripts between the PsA-R group vs. HC, with paired RefSeq, were included. Then, gene set enrichment analysis of other represented GO classes was made by fold enrichment and associated *p*-value (absolute count of identified transcripts vs. expected) for macro- and microcategories. Additionally, coding DEGs were represented in a chromosomic map to visualize their distribution. From the comparative list of DEGs in the PsA-R vs. HC condition, coding mRNAs were selected, interactions were analyzed by the STRING software (free version, V 10.5), and biological functions and annotations were determined by Gene Ontology.

### 2.5 RT-qPCR validation analysis

Twelve genes were selected from the abovementioned analysis considering literature, GO, and STRING data results and included in the validation phase completed in a larger PsA cohort (39 PsA-R + 40 PsA-A) and 40 HCs.

Extracted RNAs from whole blood were quantified by Qubit 3.0 fluorometer (Thermo Fisher Scientific, Waltham, MA, USA) and retrotranscribed by a High-Capacity RNA-cDNA kit (Invitrogen, Vilnius, Lithuania). The qPCR reactions were prepared in a final volume of 10 µL, with 5 µL of 2× TaqMan Fast Advanced Master Mix (Applied Biosystems, Foster City, CA, USA), 0.5 µL of each 20× primer, and 1 µL of sample (5 ng of cDNA template per reaction). Thermal profiling consisted of a first cycle at 50°C for 2 min, a second cycle at 95°C for 2 min, followed by 40 cycles of amplification at 95°C for 1 s and 60°C for 20 s. qPCR reactions were run in triplicate on a thermal cycler StepOne Plus (Applied Biosystems, Foster City, CA, USA).

Gene expression was measured using the following TaqMan Gene Expression Assay primers (Applied Biosystems, Foster City, CA, USA): *FCAR* (Hs02572026_s1), *CEACAM8* (Hs00266198_m1), *FOS* (Hs04194186_s1), *BPI* (Hs01552756_m1), *DEFA1B* (Hs07287122_m1), *ANPEP* (Hs00174265_m1), *ALPL* (Hs01029144_m1), *CHI3L1* (Hs01072228_m1), *PADI2* (Hs01042505_m1), *KLRB1* (Hs00174469_m1), *CCD50* (Hs01047000_m1), and *TNSF14* (Hs00542476_g1). Glyceraldehyde 3 phosphate dehydrogenase (*GAPDH*) was used as the housekeeping gene (Hs02758991_g1).

Gene expression quantification was made by the 2^−ΔΔCt^ method for relative quantification (RQ), and the fold change (FC) cutoff was ±1.5 for RQ comparative analysis between groups.

### 2.6 Statistical analysis

Categorical variables were expressed as absolute values and frequencies (%). Normally and non-normally distributed continuous variables were reported as the mean ± standard deviation (SD) and median and IQR, respectively. Student’s *t*-test was applied in the validation phase to compare the mean relative quantification values in the three study groups. The following comparisons were performed: PsA-R vs. HC, PsA-R vs. PsA-A, and PsA-A vs. HC. A *p*-value of <0.05 was considered statistically significant.

## 3 Results

### 3.1 Transcriptomic profiling of the remission state

**Figure 1:**
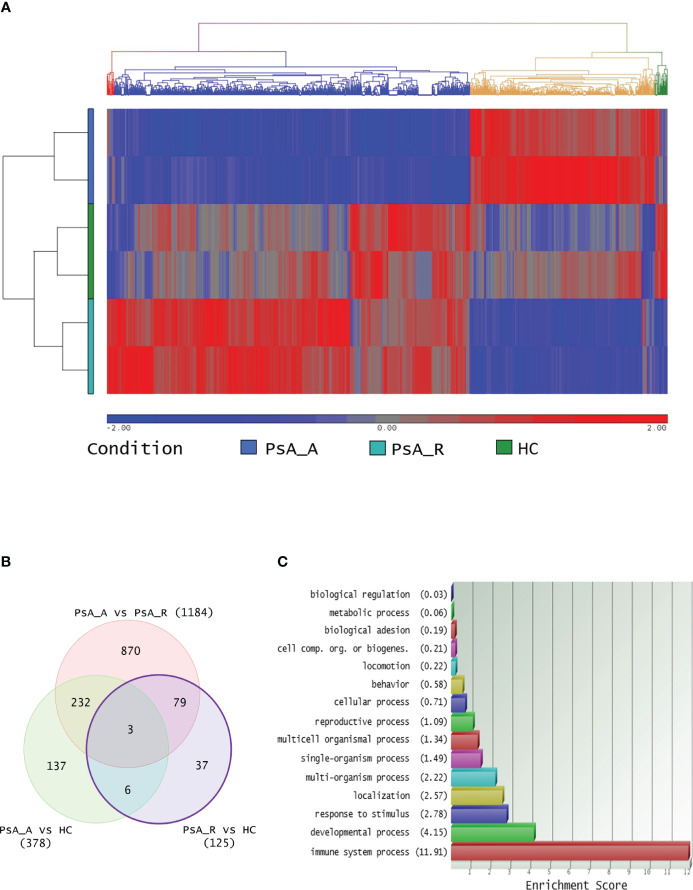
**(A)** Transcriptomic analysis and clustering. Hierarchical clustering of 1,364 differentially expressed transcripts in at least one comparison in analysis (FC 1.5 and *p*-value 0.05). The profiled conditions are in the rows (in duplicates, two rows/condition. PsA_A, active psoriatic arthritis; PsA_R, remission psoriatic arthritis; HCs, healthy controls), and the transcripts are in the columns by a pseudocolor scale with expression values normalized to zero, SD = 1 (blue, lower abundance; red, higher abundance), as indicated in the legend scale. Four clusters are represented by four colors in the upper dendrogram, suggesting that these conditions have distinct signatures (or similarities). **(B)** Venn diagram illustrating the overlap between 1,364 transcripts differentially expressed on three comparative lists (identified with a minimum HR of 1.5 and *p-*value 0.05, no FDR correction applied). **(C)** DEG Gene Ontology analysis. For the bioinformatics Gene Ontology (GO) analysis, only differentially expressed transcripts between PsA-R condition vs. HC, with paired RefSeq, were included. The enrichment analysis about more represented GO classes (histogram bars) was made by fold enrichment and associated *p*-value (the reported enrichment score in brackets is the absolute count of identified transcripts vs. expected), both for macro- and microcategories.

More than 1,000 transcripts differentially expressed in at least one of the three comparisons were identified. The hierarchical clustering with heatmap is reported in **Figure 1A**. In particular, 125 DEGs (65 up- and 60 downregulated) were identified comparing the PsA-R vs. the HC group, 1,184 (753 up- and 431 downregulated) comparing the PsA-R vs. the PsA-A group, and 378 (378 up- and 314 downregulated) comparing the PsA-A vs. the HC group. The numbers of DEGs for each comparison and the respective overlaps are represented in the Venn diagram in **Figure 1B**, and the complete list of all DEGs identified in the comparison object of this study is reported in **Supplementary Material 1**.

### 3.2 Biological function analysis of DEGs in clinical remission

The bioinformatics gene set enrichment analysis by the GO software of the 125 DEGs identified in the PsA-R vs. the HC comparison showed that they were primarily involved in the “immune system processes” (**Figure 1C**). A subanalysis on “microcategories” of the immune system process related to the DEGs is reported in **Supplementary Material 2**.

**Figure 2:**
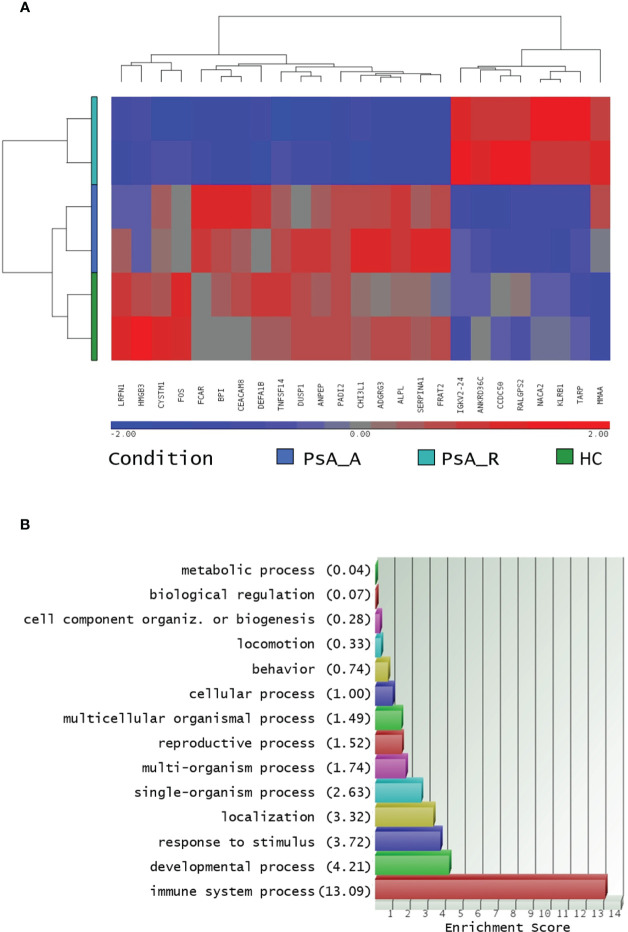
**(A)** Hierarchical clustering of coding DEGs in remission state. Heatmap of 24 filtered coding DEGs in the PsA remission state and their expression in all profiled conditions, analysis in biological duplicates (cutoff FC ± 1.5, *p*-value 0.05). The profiled conditions are in the rows (in duplicates, two rows/condition: PsA_A, active psoriatic arthritis; PsA_R, remission psoriatic arthritis; HCs, healthy controls), and the DEGs are in the columns by a pseudocolor scale with expression values normalized to zero, SD = 1 (blue, lower abundance; red, higher abundance), as indicated in the legend scale. **(B)**: coding DEGs Gene Ontology analysis. Only coding DEGs in the PsA-R condition vs. HC, with paired RefSeq, were included. The enrichment analysis about more represented GO classes (histogram bars) was made by fold enrichment and associated *p*-value (the reported enrichment score in brackets is the absolute count of identified transcripts vs. expected).

**Figure 3:**
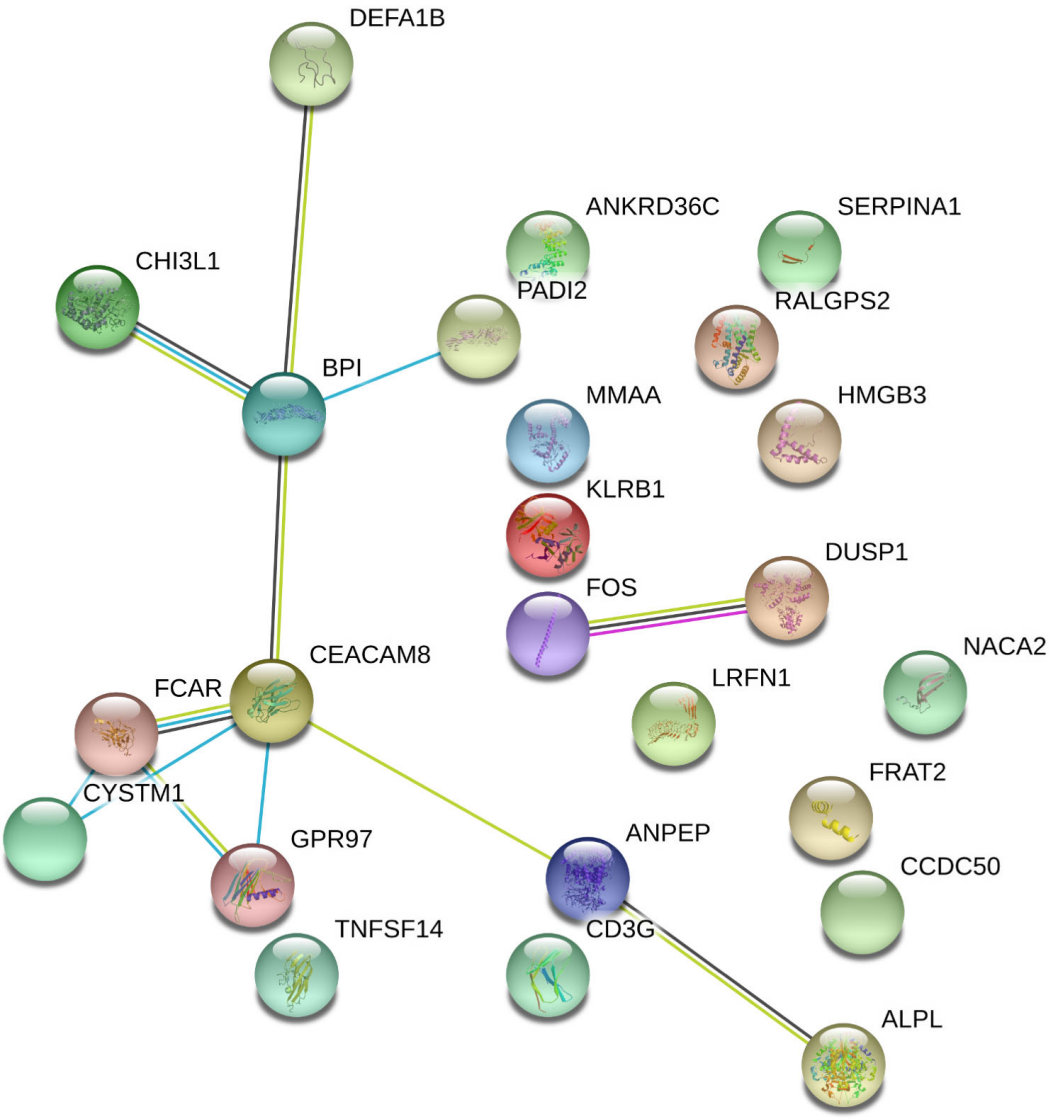
Interactome of coding DEGs misregulated on PsA clinical remission. Differentially expressed genes (DEGs) in the remission (PsA-R) group vs. healthy controls (HCs) analyzed for multiple protein interactions by the STRING software V10.5 (raw data and coordinates in **Supplementary Material 4**). Network nodes representing proteins, splice isoforms, or post-translational modifications are collapsed, i.e., each node represents all the proteins produced by a single, protein-coding gene locus. Edges represent protein–protein associations and are drawn as follows: red line = presence of fusion evidence; green line = neighborhood evidence; blue line = cooccurrence evidence; purple line = experimental evidence; yellow line = textmining evidence; light blue line = database evidence; black line = coexpression evidence. Edge associations are meant to be specific and meaningful, i.e., proteins jointly contribute to a shared function; this does not necessarily mean they are physically binding to each other. Represented network stats: number of nodes = 24; number of edges = 12; average node degree = 1; avg. local clustering coefficient = 0.369; expected number of edges = 3; PPI enrichment *p*-value = 3.17e−05.

Out of the 125 DEGs identified by comparing the PsA-R vs. the HC group, only 25 were coding genes. Thus, according to the preset methodology, they were selected for the in-depth functional and biological analysis. The respective hierarchical clustering with the heatmap is reported in **Figure 2A**, and similar to the previous GO analysis, the biological function study of the 25 coding DEGs demonstrated their primary involvement in the “immune system processes” (**Figure 2B**; for the complete list of symbols and annotated functions by the Partek software, see **Supplementary Material 3**). Moreover, when such DEGs were further analyzed for functions and interactions, the bioinformatics STRING software tool built an interaction network between 24 putative proteins, with more significant interactions than expected (**Figure 3**). For the STRING raw data analysis, legend, settings, and results, see **Supplementary Material 4**. Lastly, in **Supplementary Material 5**, the karyomap figure shows these genes’ chromosomic mapping.

### 3.3 Extended gene expression analysis of coding DEGs in the remission state

Matching our bioinformatics data with the current evidence regarding the 25 coding DEGs identified in the PsA-R vs. the HC comparison, 12 were selected for the validation phase by gene expression quantification. Their symbols, RefSeq, annotated functions, and chromosomic position are described in **Table 2**.

**Table 2:** All validated DEGs listed for their gene symbol, full name, synonyms, cytoband, and functions annotated by RefSeq and UniProt sources.

Gene symbol	Full name	Synonyms	Annotated functions	Cytoband
***CCDC50***	Coiled-coil domain containing 50	YMER; C3orf6; DFNA44	Encodes a soluble, cytoplasmic, tyrosine-phosphorylated protein with multiple ubiquitin-interacting domains that may function as a negative regulator of NF-κB signaling and as an effector of epidermal growth factor (EGF)-mediated cell signaling.	3q28
***KLRB1***	Killer cell lectin-like receptor B1	CD161, CLEC5B, NKR, NKR-P1, NKR-P1A, NKRP1A, hNKR-P1A	Plays an inhibitory role in natural killer (NK) cell cytotoxicity. Activation results in sphingomyelinase/SMPD1 stimulation, also leads to enhanced T-cell proliferation induced by anti-CD3. Binds also to CLEC2D/LLT1 as a ligand and inhibits NK cell-mediated cytotoxicity as well as interferon-gamma secretion in target cells.	12p13.31
***ANPEP***	Alanyl aminopeptidase, membrane	APN; CD13; LAP1; P150; PEPN; GP150	Involved in the processing of various peptides including peptide hormones, angiotensins III and IV, neuropeptides, and chemokines. May also be involved in the cleavage of peptides bound to major histocompatibility complex class II molecules of antigen-presenting cells.	15q26.1
***DEFA1B***	Defensin alpha 1B	HP1; HP-1; HNP-1	Family of antimicrobial and cytotoxic peptides involved in host defense, abundant in the granules of neutrophils and also found in the epithelia of mucosal surfaces such as those of the intestine, respiratory tract, urinary tract, and vagina.	8p23.1
***BPI***	Bactericidal permeability increasing protein	rBPI; BPIFD1	Belongs to the BPI/LBP/Plunc superfamily. The cytotoxic action of BPI is limited to many species of Gram-negative bacteria.	20q11.23
***CHI3L1***	Chitinase 3 like 1	ASRT7, CGP-39, CHI3L1, CHIL1, GP-39, GP39, HC-GP39, HCGP-39, HCGP-3P, YKL-40, YKL40, YYL-40	Chitinases catalyze the hydrolysis of chitin. The protein is secreted by activated macrophages, chondrocytes, neutrophils, and synovial cells. The protein is thought to play a role in the process of inflammation and tissue remodeling.	1q32.1
***FOS***	Fos proto-oncogene, AP-1 transcription factor subunit	AP-1, C-FOS, p55	Nuclear phosphoprotein forms a complex with the JUN/AP-1 transcription factor with an important role in signal transduction, cell proliferation, and differentiation. Forms a multimeric SMAD3/SMAD4/JUN/FOS complex at the AP1/SMAD-binding site to regulate TGF-beta-mediated signaling. Has a critical function in regulating the development of cells destined to form and maintain the skeleton notably involved in the osteoclastogenesis by RANK ligand signaling, in inflammatory bone and skin disease.	14q24.3
***ALPL***	Alkaline phosphatase, biomineralization associated	HOPS; TNAP; TNALP; APTNAP; TNSALP; AP-TNAP	Encodes a member of the family of phosphatases: intestinal, placental, placental-like, and liver/bone/kidney. The mature enzyme may play a role in bone mineralization. Mutations in this gene have been linked to hypophosphatasia, a disorder that is characterized by hypercalcemia and skeletal defects.	1p36.12
***PADI2***	peptidyl arginine deiminase 2	MKIAA0994, PAD-H19, PAD2, PADI2, PDI, PDI2	Encodes a member of the family of enzymes, which catalyze the post-translational deimination of proteins by converting arginine residues into citrullines. Known substrates for this enzyme include vimentin in skeletal muscle and macrophages.	1p36.13
***TNFSF14***	TNF superfamily member 14	LTg; CD258; HVEML; LIGHT	The protein encoded is a member of the tumor necrosis factor (TNF) ligand family: a cytokine ligand for TNFRSF14 may function as a costimulatory factor for the activation of lymphoid cells and as a deterrent to infection by herpesvirus. This protein has been shown to stimulate the proliferation of T cells and trigger apoptosis of various tumor cells.	19p13.3
***FCAR***	Fc fragment of IgA receptor	CD89; FcalphaRI; CTB-61M7.2	This gene encodes a receptor for the Fc region of IgA, a transmembrane glycoprotein present on the surface of myeloid lineage cells where it mediates immunologic responses to pathogens and stimulation of the release of inflammatory mediators.	19q13.42
***CEACAM8***	CEA cell adhesion molecule 8	CD67; CGM6; CD66b; NCA-95	Belongs to the immunoglobulin superfamily. Cell surface glycoprotein that plays a role in cell adhesion. Heterophilic interaction with CEACAM8 occurs in activated neutrophils.	19q13.2

The RT-qPCR validations in the large PsA cohort (39 PsA-R + 40 PsA-A patients) and 40 HCs measured the expression of all selected genes in all subjects as shown in **Table 3**.

**Table 3:** Gene expression quantification of DEGs on PsA remission state.

	FC microarray	FC RT-qPCR (*p*-value)		
**DEG**	**PsA-R vs. HC**	**PsA-R vs. HC**	**PsA-R vs. PsA-A**	**PsA-A vs. HC**
***FCAR***	−1.56	−1.1 (*0.362*)	−1.3 (*0.114*)	1.1 (*0.295*)
***CEACAM8***	−2.3	−1.2 (*0.542*)	−1.5 (*0.241*)	1.2 (*0.542*)
***FOS***	−1.51	**−2.0 (*0.005*)**	−1.4 (*0.150*)	-1.5 (*0.103*)
***BPI***	−1.57	−1.1 (*0.596*)	−1.5 (*0.151*)	1.27 (*0.400*)
***DEFA1B***	−2.3	−1.4 (*0.366*)	−2.7 (*0.067*)	1.9 (*0.170*)
***ANPEP***	−1.68	1.0 (*0.618*)	−1.1 (*0.220*)	1.1 (*0.065*)
***ALPL***	−1.7	−1.1 (*0.763*)	−1.25 (*0.035*)	1.3 (*0.120*)
***CCD50***	1.5	**1.5 (*0.005*)**	1.8 (*<0.001*)	−1.25 (*0.006*)
***PADI2***	−1.54	−1.1 (*0.631*)	−1.1 (*0.388*)	1.0 (*0.773*)
***KLRB1***	1.52	−1 (*0.184*)	1.6 (*<0.001*)	−1.6 (*0.001*)
***CHI3L1***	−1.59	−1.1 (*0.425*)	−1.0 (*0.809*)	−1.0 (*0.595*)
***TNSF14***	−1.51	1.1 (*0.157*)	−1.1 (*0.213*)	1.2 (*0.027*)

In the gray column, the fold change (FC) microarray values of differentially expressed genes (DEGs) in the remission state (PsA-R) vs. healthy condition (HC); in the green column, the same DEGs validated by the RT-qPCR technique [TaqMan chemistry, 2^−ΔΔCt^ method for relative quantification (RQ)]; in the white columns, the FC values of DEGs in the other comparisons. Differential analysis between groups (39 PsA-R vs. 40 HC vs. 40PsA-active) was made by fold change (FC) cutoff ±1.5 to estimate gene dysregulation (overexpressed ≥1.5; −1.5 ≥ downregulated); the p-value cutoff for significance is ≤0.05.

**Figure 4:**
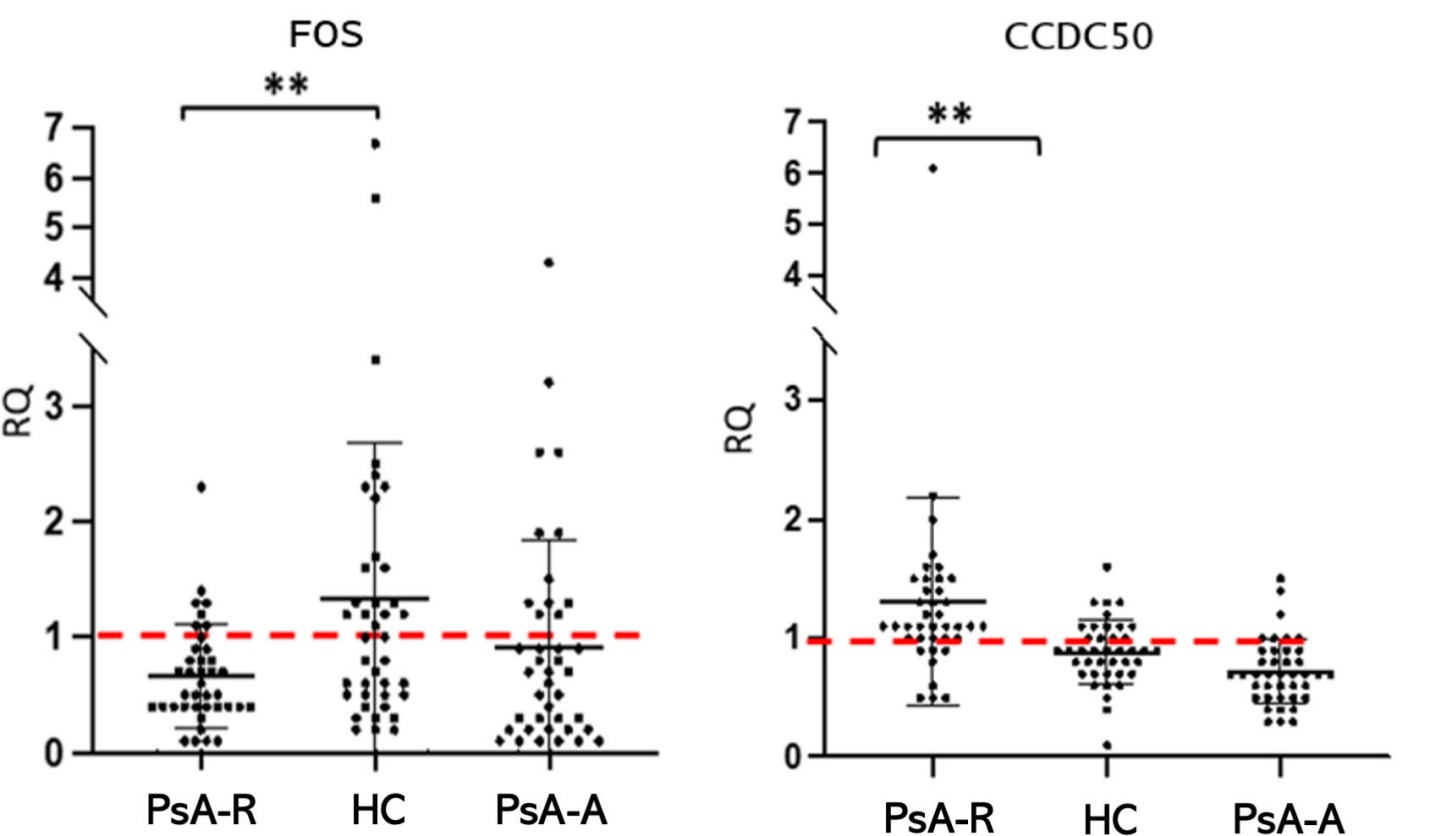
The *FOS* and *CCDC50* dysregulation in the PsA clinical remission. Mean (SD) of relative quantification (RQ) of *FOS* and *CCDC50* genes in the remission (PsA-R), active (PsA-A), and healthy condition (HC). Analysis by the 2^−ΔΔCt^ method. **p-value = 0.005.

In the clinical remission vs. healthy condition comparison, data obtained in the single-gene expression dosage significantly confirmed the downregulation (FC −2.0; *p* 0.005) of *FOS* and the upregulation (FC + 1.5; *p* 0.005) of *CCDC50* (alias *YMER*) genes in the PsA-R state (**Figure 4**). For further analysis, we evaluated the association between CRP levels and RQ values of both these genes, demonstrating a significant negative and positive correlation, respectively, with *CCDC50* [Pearson’s correlation coefficient (*r*): −0.240; *p* = 0.035] and *FOS* (*r*: 0.386; *p* = 0.001).

The differential analysis between groups also showed a significative misregulation of other genes in other comparisons (PsA-R vs. PsA-A; PsA-A vs. HC) (see **Table 3**). In particular, there was a significative downregulation of *KLRB1* (FC −1.6; *p* 0.001) in the active disease vs. healthy condition, while the comparison between the remission vs. active PsA exhibited the overexpression of *CCDC50* (FC 1.8; *p* < 0.001) and *KLRB1* (FC 1.6; *p* < 0.001) (RQ and FC values of all validated DEGs are shown in **Table 3**).

## 4 Discussion

This is the first gene expression study specifically designed to explore the molecular mechanisms underlying the clinical remission in PsA patients through an investigative approach primarily based on comparing the remission and the healthy condition.

The comparative transcriptomic analysis showed that clinical remission was similar but not identical to the healthy state. Indeed, the presence of 125 DEGs suggests that the TNFi-induced clinical remission is not synonymous with molecular disease inactivation leading to a “back to a healthy state,” but it is a condition characterized by several misregulated transcripts that, on the one hand, may represent the persistence of underlying disease activity and, on the other hand, may mean the activation of mechanisms sustaining disease remission.

The subsequent phase of bioinformatics analysis showed that the coding DEGs in clinical remission were strictly correlated to each other in a strong interaction network and were primarily involved in functions related to immune system processes. These *in-silico* predictions were confirmed by the validation phase of this study, where the RT-qPCR single-gene expression analysis showed in all profiled conditions the dysregulation of genes strictly involved in inflammatory and immune processes.

The primary analysis of this study, based on comparing the clinical remission with the healthy condition, revealed the down- and upregulation of *FOS* and *CCDC50* genes, respectively, which are both reported as having a significant role in the inflammatory process and osteoclastogenesis.

*FOS* (Fos Proto-Oncogene, AP-1 Transcription Factor Subunit) is a protein-coding gene. The *FOS* gene family consists of four members: *FOS, FOSB, FOSL1*, and *FOSL2*. These genes encode leucine zipper proteins that can dimerize with proteins of the JUN family, thereby forming the transcription factor complex AP-1 (19). After being induced by several extra- and intracellular stimuli, the immediate early gene product Fos translates into the regulation of downstream target genes implicated in various cellular processes, including inflammatory response and osteoclastogenesis regulation (20). In this regard, it has been described how Fos/AP-1 has an important role in the induction of NFAT-dependent genes coding many cytokines such as IL-2 and IL-3, granulocyte–macrophage colony-stimulating factor, IL-4, IL-5, IL-13, IFNγ, TNFα, CD40L, FasL, CD5, Igκ, CD25, and the chemokines IL-8 and MIP1α (21). Furthermore, previous studies reported that AP-1 could affect the severity of inflammation through other mechanisms, including the regulation of naive T-cell differentiation into T helper 1 (Th1) or Th2 cells and modulation of the activity of the innate immune system (22–24).

Aside from its role in the inflammatory process, there is much evidence that *FOS* has an important role in the regulation of osteoclastogenesis by RANK ligand signaling (25, 26), which in turn is demonstrated to have a crucial role in developing joint/bone destructive lesions in inflammatory arthropathies, such as rheumatoid arthritis and psoriatic arthritis (27). After binding with its receptor, RANKL triggers a signaling cascade leading to the activation of key transcription factors such as NF-κB and Fos, leading to the expression of osteoclast-specific target genes (25, 26). In particular, activation of RANKL/Fos is required for the expression of Nuclear Factor for activation of T cells c1 (NFATc1) and interferon-b (IFN-b), two critical actors in osteoclast differentiation (28, 29). Notably, AP-1 activity can also affect the severity of primary arthritis with mechanisms different from the regulation of osteoclastogenesis, such as induction of MMP production (30).

A scarce amount of data is available on the potential role of *FOS* in PsA. Interestingly, data are available on rheumatoid arthritis (RA), where previous studies reported that Fos/AP-1 and interleukin 1β (IL-1β) influence each other’s gene expression and activity, resulting in an orchestrated cross-talk that, in turn, seems to have an important role in the accrual of joint damage in experimental RA models characterized by the enhancement of Fos/AP-1 activity. For this purpose, Yukiko et al. designed and synthesized a selective inhibitor of Fos/AP-1 to resolve arthritis in a mouse model of the RA disease (31).

Our analysis shows a downregulation of *FOS* in remission and active PsA patients vs. healthy controls (remission < active < healthy), suggesting that such misregulation may occur to counterbalance its pro-inflammatory and pro-osteoclastogenic functions. The fact that *FOS* is slightly downregulated in PsA-A vs. HC could suggest that this mechanism is also established in patients with active disease, but not sufficiently to maintain homeostasis.

*CCDC50* (alias *YMER*) encodes a soluble, cytoplasmic, tyrosine-phosphorylated protein with multiple ubiquitin-interacting domains that may be multifunctional in several signaling pathways (32). *CCDC50* overexpression attenuates NF-κB, a critical regulator of innate and adaptive immunity, in collaboration with A20 deubiquitinase (33), that, in turn, plays an important role in the termination of NF-κB signaling and the resolution of inflammation. In particular, *CCDC50* harbors a ubiquitin-binding domain (UBD) that may act as an adaptor molecule for A20, a mechanism important for NF-κB inhibition (33). In fact, the ubiquitin-modifying protein A20 is a broadly expressed cytoplasmic protein induced by TNFα stimulation, and it has been identified as an inhibitor of TNF-induced NF-κB activation or apoptosis. In the literature, it is established that A20 is a critical negative regulator of NF-κB (34), and A20-deficient cells fail to terminate TNF-induced NF-κB signaling (35). Furthermore, *CCDC50* was identified as a gene whose expression is highly decreased in osteoclastogenesis upon myostatin treatment *in vitro*. It could inhibit the function of myostatin in osteoclastogenesis by blocking NF-κB and MAPK pathways. In this model, overexpression of *CCDC50* diminishes NF-κB signaling, whereas knockdown of endogenous *CCDC50* upregulates NF-κB signaling, suggesting that CCD50 functions as a negative regulator for NF-κB signaling (36).

To our knowledge, no specific data are currently available on the role of *CCDC50* in PsA. In affected patients in clinical remission from our cohort, the upregulation of *CCDC50* could have a protective role, contributing to bone homeostasis recovery and avoiding the most aggressive disease outcome, such as articular erosion, by inhibiting the osteoclastogenesis process. This assumption is further supported by the demonstration that *CCDC50* is downregulated in active patients compared with those in clinical remission. Therapeutic strategies targeting *CCDC50* may be conducive to treating diseases related to its aberrant expression.

Taken together, the opposite function of the two inversely misregulated genes, *FOS* and *CCDC50*, in PsA patients in clinical remission would confirm that the former did not represent “a back to the healthy condition,” but it is probably the result of a new balance between inhibition of pro-inflammatory and pro-osteoclastic processes and enhancement of protective mechanism against the same inflammatory and osteogenic phenomena. In particular, clinical remission might be molecularly driven by *FOS*gene downregulation, determining the minor activation of RANKL and subsequently slight osteoclastogenesis, and *CCDC50* overexpression, which imply major NF-κB inhibition and type I IFN pathway restriction. No data from the literature showed direct interactions between the products of these two genes; however, a cooperation cannot be excluded because of their involvement in the same downstream pathway. In this regard, bioinformatics tools such as the Gene Transcription Regulation Database (GTRD, website http://gtrd20-06.biouml.org/) allow us to hypothesize that the *CCDC50* gene sequence may be a possible target of the Fos transcription factor; however, further studies need to be conducted for this purpose.

The secondary analysis of our study comparing the remission with active disease, and the latter with healthy controls, showed that there was, as expected, a significant difference in terms of the transcriptomic profile also between TNFi-induced remission and the active disease, as well as between the active disease and the healthy condition. The DEG validation extended to these further comparisons showed the misregulation of *DEFA1B* and *KLRB1* (PsA-A vs. HC) and *BPI, CEACAM8, DEFA1B*, and *KLRB1* (PsA-R vs. PsA-A), respectively. However, these comparisons were not the primary objective of this study and deserve further, separate, in-depth investigation.

This work provides previous unreported information on the molecular characterization of the clinical remission in PsA, describing for the first time in this condition the dysregulation of two key genes notably involved in inflammatory and bone metabolism processes. These findings pave the way into a research field that is of clinical interest and provide data to the debate about considering remission as a condition with molecular disease inactivation leading to a “back to a healthy state.” In the precision medicine era, more molecular data about PsA disease activity assessment are needed: in the near future, the biomarker discovery of a molecular remission state achievement, for a better and precise assessment of the actual major goal of PsA management, should be improved.

This study has some limitations. First, the enrolment of PsA patients in clinical remission solely induced by TNFi prevents a generalization of the results of the remission induced by other treatments. However, this methodological choice assumed that different treatments might induce different molecular mechanisms sustaining remission. Thus, for this reason, a homogeneous PsA cohort in remission with the most widely used first-line bioDMARD was selected (37). It is noteworthy that remission induced by TNFi is particularly consistent with the result of our study and corroborates their validity, as both *FOS* and *CCD50* are involved in the regulation of pathways where TNF has a key role. Second, in the validation phase of the study, only the coding transcripts were evaluated, aiming to assess the interaction between gene products in existing biological pathways and processes. Finally, in the RT-qPCR validation phase, 12 coding genes out of 24 mRNAs were evaluated. Although this selection was based on previous *in-silico* investigations (GO gene set enrichment and STRING software) and an in-depth literature review, also the remaining genes potentially may have a role in sustaining the TNFi remission, deserving further research.

To date, no successful models of TNFi prediction in PsA are available clinically (38), and the research field of biomarker discovery related to molecular remission achievement is only at an early stage (39–41). Whole-blood transcriptomic profiling performed in this study suggests that TNFi-induced clinical remission in PsA is similar to a healthy condition, but not identical, differing for a list of 125 transcripts and particularly for the *FOS* and *CCDC50* gene expression amount. The molecular characterization of PsA disease activity may have a crucial role in identifying biological as well as clinical remission, favoring a more effective application of the prospective treat-to-target strategy.

## Data availability statement

The original contributions presented in the study are included in the article/**Supplementary Material**. Further inquiries can be directed to the corresponding author.

## Ethics statement

The studies involving humans were approved by the local ethical committee (PG/2018/16313; 12th November 2018). The studies were conducted in accordance with the local legislation and institutional requirements. The participants provided their written informed consent to participate in this study.

## Author contributions

MMA: Conceptualization, Data curation, Formal Analysis, Investigation, Methodology, Software, Validation, Writing – original draft. AF: Data curation, Formal Analysis, Investigation, Methodology, Writing – original draft. IC: Data curation, Formal Analysis, Investigation, Methodology, Writing – review & editing. MC: Data curation, Formal Analysis, Investigation, Writing – review & editing. EC: Data curation, Formal Analysis, Investigation, Writing – review & editing. MNR: Data curation, Formal Analysis, Investigation, Writing – review & editing. MP: Data curation, Formal Analysis, Investigation, Writing – review & editing. AC: Conceptualization, Data curation, Formal Analysis, Supervision, Validation, Writing – original draft.
