# Supplementary material for: Molecular profiling of clinical remission in psoriatic arthritis reveals dysregulation of FOS and CCDC50 genes: a gene expression study
Source: Front Immunol. 2023 Oct 27;14:1274539. doi: 10.3389/fimmu.2023.1274539 (PMC10641465; doi:10.3389/fimmu.2023.1274539)
Supplement: Supplementary file 1 [file DataSheet_1.docx]

**Supplementary Material 1. Complete list of DEGs of PsA_R vs HC condition.** The complete list of all 125 Differentially Expressed Genes (DEGs) identified in the Remission state vs healthy condition comparison is reported.
