# Supplementary material for: Molecular profiling of clinical remission in psoriatic arthritis reveals dysregulation of FOS and CCDC50 genes: a gene expression study
Source: Front Immunol. 2023 Oct 27;14:1274539. doi: 10.3389/fimmu.2023.1274539 (PMC10641465; doi:10.3389/fimmu.2023.1274539)

**Supplementary Material 2. DEGs associated with the GO microcategory "Immune System Process".** In the Forestplots are represented: in the abscissa the number of transcripts included, in pseudocolors the direction of the regulation (downregulated = red; upregulated = green) in the comparison under analysis (Panel A: PsA Remission vs HC; Panel B: PsA Active vs HC;).


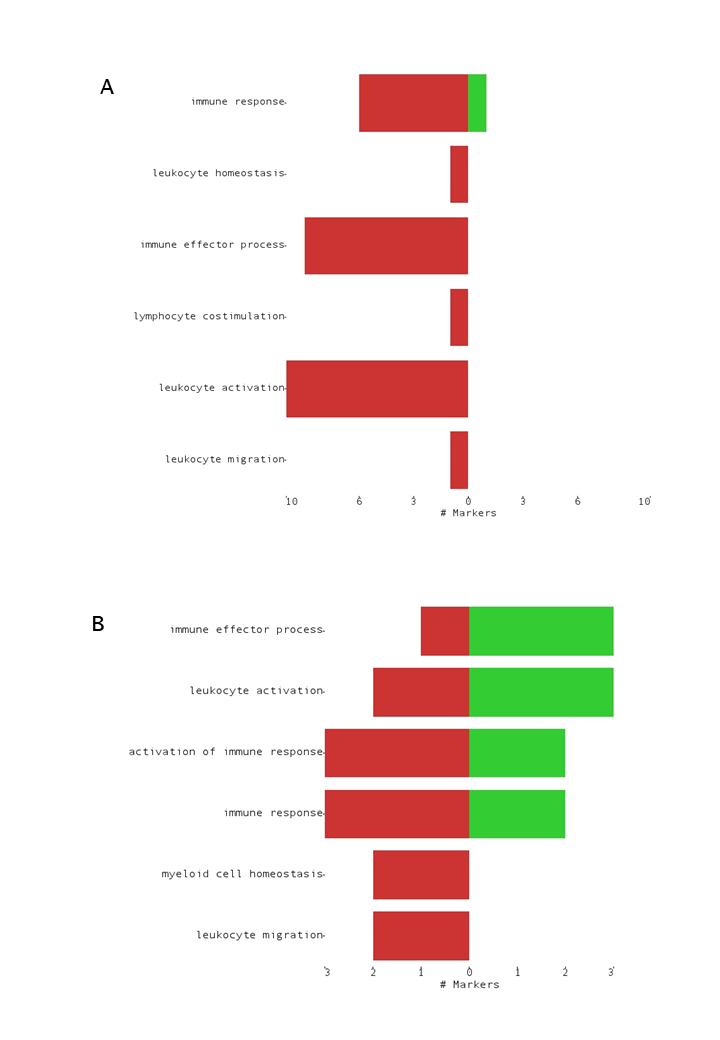

Supplement: Supplementary file 2 [file DataSheet_2.docx]
