# Supplementary material for: Molecular profiling of clinical remission in psoriatic arthritis reveals dysregulation of FOS and CCDC50 genes: a gene expression study
Source: Front Immunol. 2023 Oct 27;14:1274539. doi: 10.3389/fimmu.2023.1274539 (PMC10641465; doi:10.3389/fimmu.2023.1274539)
Supplement: Supplementary file 3 [file DataSheet_3.docx]

**Supplementary Material 3. Full list of DEGs symbols and annotated functions by Partek software.**
