# Supplementary material for: Molecular profiling of clinical remission in psoriatic arthritis reveals dysregulation of FOS and CCDC50 genes: a gene expression study
Source: Front Immunol. 2023 Oct 27;14:1274539. doi: 10.3389/fimmu.2023.1274539 (PMC10641465; doi:10.3389/fimmu.2023.1274539)
Supplement: Supplementary file 4 [file DataSheet_4.docx]

**Supplementary Material 4. STRING software raw data and network coordinates in tabular form.**

| **#node** | **x_position** | **y_position** | **color** | **annotation** |
| --- | --- | --- | --- | --- |
| ***CD3G*** | 0.524462 | 0.81178 | rgb(117,239,166) | T-cell surface glycoprotein CD3 gamma chain; Part of the TCR-CD3 complex present on T-lymphocyte cell surface that plays an essential role in adaptive immune response. When antigen presenting cells (APCs) activate T-cell receptor (TCR), TCR-mediated signals are transmitted across the cell membrane by the CD3 chains CD3D, CD3E, CD3G and CD3Z. All CD3 chains contain immunoreceptor tyrosine-based activation motifs (ITAMs) in their cytoplasmic domain. Upon TCR engagement, these motifs become phosphorylated by Src family protein tyrosine kinases LCK and FYN. |
| ***ANPEP*** | 0.542 | 0.739529 | rgb(0,28,178) | Aminopeptidase N; Broad specificity aminopeptidase which plays a role in the final digestion of peptides generated from hydrolysis of proteins by gastric and pancreatic proteases. Also involved in the processing of various peptides including peptide hormones, such as angiotensin III and IV, neuropeptides, and chemokines. May also be involved the cleavage of peptides bound to major histocompatibility complex class II molecules of antigen presenting cells. May have a role in angiogenesis and promote cholesterol crystallization. |
| ***CCDC50*** | 0.662923 | 0.76466 | rgb(166,239,117) | Coiled-coil domain-containing protein 50; Involved in EGFR signaling; Deafness associated genes |
| ***HMGB3*** | 0.635231 | 0.411257 | rgb(239,190,117) | High mobility group protein B3; Multifunctional protein with various roles in different cellular compartments. May act in a redox sensitive manner. Associates with chromatin and binds DNA with a preference to non- canonical DNA structures such as single-stranded DNA. Can bent DNA and enhance DNA flexibility by looping thus providing a mechanism to promote activities on various gene promoters (By similarity). Proposed to be involved in the innate immune response to nucleic acids by acting as a cytoplasmic promiscuous immunogenic DNA/RNA sensor (By similarity). |
| ***MMAA*** | 0.524462 | 0.411257 | rgb(101,199,255) | Methylmalonic aciduria type A protein, mitochondrial; GTPase, binds and hydrolyzes GTP. Involved in intracellular vitamin B12 metabolism, mediates the transport of cobalamin (Cbl) into mitochondria for the final steps of adenosylcobalamin (AdoCbl) synthesis. Functions as a G-protein chaperone that assists AdoCbl cofactor delivery from MMAB to the methylmalonyl-CoA mutase (MUT) and reactivation of the enzyme during catalysis. |
| ***DEFA1B*** | 0.420154 | 0.128534 | rgb(190,239,117) | Defensin, alpha 1B; Defensin 1 and defensin 2 have antibacterial, fungicide and antiviral activities. Has antimicrobial activity against Gram- negative and Gram-positive bacteria. Defensins are thought to kill microbes by permeabilizing their plasma membrane. |
| ***LRFN1*** | 0.579846 | 0.623298 | rgb(187,255,101) | Leucine-rich repeat and fibronectin type III domain-containing protein 1; Promotes neurite outgrowth in hippocampal neurons. Involved in the regulation and maintenance of excitatory synapses. Induces the clustering of excitatory postsynaptic proteins, including DLG4, DLGAP1, GRIA1 and GRIN1 (By similarity). |
| ***BPI*** | 0.408154 | 0.390838 | rgb(0,178,157) | Bactericidal permeability-increasing protein; The cytotoxic action of BPI is limited to many species of Gram-negative bacteria; this specificity may be explained by a strong affinity of the very basic N-terminal half for the negatively charged lipopolysaccharides that are unique to the Gram-negative bacterial outer envelope. Has antibacterial activity against the Gram-nagative bacterium P.aeruginosa, this activity is inhibited by LPS from P.aeruginosa; Belongs to the BPI/LBP/Plunc superfamily. |
| ***DUSP1*** | 0.629692 | 0.529058 | rgb(255,175,101) | Dual specificity protein phosphatase 1; Dual specificity phosphatase that dephosphorylates MAP kinase MAPK1/ERK2 on both 'Thr-183' and 'Tyr-185', regulating its activity during the meiotic cell cycle; Belongs to the protein-tyrosine phosphatase family. |
| ***RALGPS2*** | 0.607538 | 0.340576 | rgb(239,166,117) | Ras-specific guanine nucleotide-releasing factor RalGPS2; Guanine nucleotide exchange factor for the small GTPase RALA. May be involved in cytoskeletal organization. May also be involved in the stimulation of transcription in a Ras-independent fashion (By similarity). |
| ***CYSTM1*** | 0.302923 | 0.709686 | rgb(101,255,163) | Cysteine-rich and transmembrane domain-containing protein 1; Cysteine rich transmembrane module containing 1 |
| ***CHI3L1*** | 0.326 | 0.310733 | rgb(14,178,0) | Chitinase-3-like protein 1; Carbohydrate-binding lectin with a preference for chitin. Has no chitinase activity. May play a role in tissue remodeling and in the capacity of cells to respond to and cope with changes in their environment. Plays a role in T-helper cell type 2 (Th2) inflammatory response and IL-13-induced inflammation, regulating allergen sensitization, inflammatory cell apoptosis, dendritic cell accumulation and M2 macrophage differentiation. Facilitates invasion of pathogenic enteric bacteria into colonic mucosa and lymphoid organs. Mediates activation of AKT1 signaling. |
| ***FOS*** | 0.524462 | 0.555759 | rgb(150,101,255) | Proto-oncogene c-Fos; Nuclear phosphoprotein which forms a tight but non- covalently linked complex with the JUN/AP-1 transcription factor. In the heterodimer, FOS and JUN/AP-1 basic regions each seems to interact with symmetrical DNA half sites. On TGF-beta activation, forms a multimeric SMAD3/SMAD4/JUN/FOS complex at the AP1/SMAD- binding site to regulate TGF-beta-mediated signaling. Has a critical function in regulating the development of cells destined to form and maintain the skeleton. It is thought to have an important role in signal transduction and cell proliferation. |
| ***ANKRD36C*** | 0.524462 | 0.269895 | rgb(141,239,117) | Ankyrin repeat domain-containing protein 36C; Ankyrin repeat domain containing |
| ***SERPINA1*** | 0.635231 | 0.269895 | rgb(117,239,117) | Alpha-1-antitrypsin; Inhibitor of serine proteases. Its primary target is elastase, but it also has a moderate affinity for plasmin and thrombin. Irreversibly inhibits trypsin, chymotrypsin and plasminogen activator. The aberrant form inhibits insulin-induced NO synthesis in platelets, decreases coagulation time and has proteolytic activity against insulin and plasmin; Belongs to the serpin family. |
| ***GPR97*** | 0.394308 | 0.744241 | rgb(239,117,117) | Adhesion G protein-coupled receptor G3; Orphan receptor that regulates migration of lymphatic endothelial cells in vitro via the small GTPases RhoA and CDC42. Regulates B-cell development (By similarity). Seems to signal through G-alpha(q)-proteins. |
| ***ALPL*** | 0.654615 | 0.879319 | rgb(239,239,117) | Alkaline phosphatase, tissue-nonspecific isozyme; This isozyme may play a role in skeletal mineralization; Belongs to the alkaline phosphatase family |
| ***PADI2*** | 0.494 | 0.332723 | rgb(215,239,117) | Protein-arginine deiminase type-2; Catalyzes the deimination of arginine residues of proteins. |
| ***TNFSF14*** | 0.413692 | 0.81178 | rgb(117,239,190) | Tumor necrosis factor ligand superfamily member 14; Cytokine that binds to TNFRSF3/LTBR. Binding to the decoy receptor TNFRSF6B modulates its effects. Activates NFKB, stimulates the proliferation of T-cells, and inhibits growth of the adenocarcinoma HT-29. Acts as a receptor for Herpes simplex virus. |
| ***NACA2*** | 0.690615 | 0.599738 | rgb(117,239,141) | Nascent polypeptide-associated complex subunit alpha-2; Prevents inappropriate targeting of non-secretory polypeptides to the endoplasmic reticulum (ER). Binds to nascent polypeptide chains as they emerge from the ribosome and blocks their interaction with the signal recognition particle (SRP), which normally targets nascent secretory peptides to the ER. Also reduces the inherent affinity of ribosomes for protein translocation sites in the ER membrane (M sites) (By similarity) |
| ***FCAR*** | 0.334308 | 0.631152 | rgb(239,141,117) | Immunoglobulin alpha Fc receptor; Binds to the Fc region of immunoglobulins alpha. Mediates several functions including cytokine production. |
| ***KLRB1*** | 0.524462 | 0.481937 | rgb(255,0,0) | Killer cell lectin-like receptor subfamily B member 1; Plays an inhibitory role on natural killer (NK) cells cytotoxicity. Activation results in specific acid sphingomyelinase/SMPD1 stimulation with subsequent marked elevation of intracellular ceramide. Activation also leads to AKT1/PKB and RPS6KA1/RSK1 kinases stimulation as well as markedly enhanced T-cell proliferation induced by anti-CD3. Acts as a lectin that binds to the terminal carbohydrate Gal-alpha(1,3)Gal epitope as well as to the N-acetyllactosamine epitope. Binds also to CLEC2D/LLT1 as a ligand and inhibits NK cell. |
| ***CEACAM8*** | 0.402615 | 0.606021 | rgb(178,171,0) | Carcinoembryonic antigen related cell adhesion molecule family; Belongs to the immunoglobulin superfamily. CEA family |
| ***FRAT2*** | 0.649077 | 0.693979 | rgb(239,215,117) | GSK-3-binding protein FRAT2; Positively regulates the Wnt signaling pathway by stabilizing beta-catenin through the association with GSK-3. |
