# Supplementary material for: Molecular profiling of clinical remission in psoriatic arthritis reveals dysregulation of FOS and CCDC50 genes: a gene expression study
Source: Front Immunol. 2023 Oct 27;14:1274539. doi: 10.3389/fimmu.2023.1274539 (PMC10641465; doi:10.3389/fimmu.2023.1274539)

**Supplementary Material 5. Karyomap of coding DEGs in the Remission state.** Chromosomic mapping of 24-filtered coding DEGs in the PsA clinical remission.


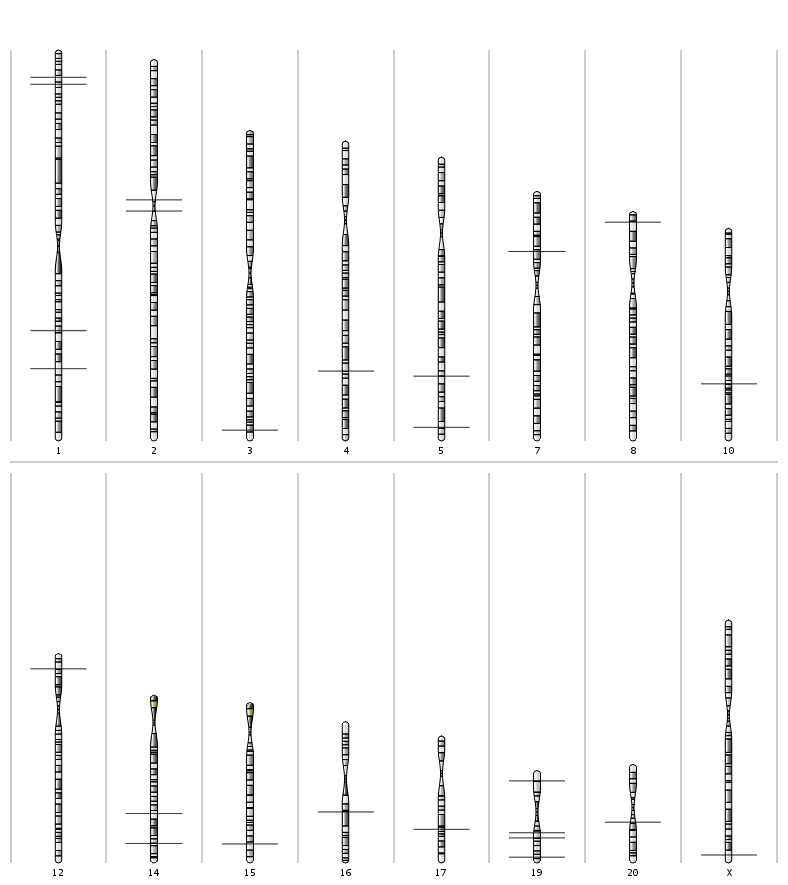

Supplement: Supplementary file 5 [file DataSheet_5.docx]
